# Infection of Rodents by *Orientia tsutsugamushi*, the Agent of Scrub Typhus, in Relation to Land Use in Thailand

**DOI:** 10.3390/tropicalmed2040053

**Published:** 2017-10-06

**Authors:** Kittipong Chaisiri, Jean-François Cosson, Serge Morand

**Affiliations:** 1 Department of Helminthology, Faculty of Tropical Medicine, Mahidol University, Bangkok 10400, Thailand; kittipong.cha@mahidol.ac.th; 2 NRA, Biologie Moléculaire et Immunologie Parasitaires et Fongiques, BIPAR, Ecole Nationale Vétérinaire d’Alfort, 94704 Maisons-Alfort Cedex, France; cosson@supagro.inra.fr; 3 CNRS ISEM-CIRAD ASTRE, Faculty of Veterinary Technology, Kasetsart University, Bangkok 10220, Thailand

**Keywords:** *Orientia tsutsugamushi*, scrub typhus, 16S rDNA amplicon sequencing, rodents, land use land cover, Thailand

## Abstract

The relationship between land use structures and occurrence of the scrub typhus agent, *Orientia tsutsugamushi,* in small wild mammals was investigated in three provinces of Thailand: Buriram, Loei, and Nan. *O. tsutsugamushi* detection was performed using 16S ribosomal DNA (rDNA) amplicon sequencing approach using Miseq Illumina platform. In total, 387 animals (rodents and shrews) were examined for the infection. The 16S rDNA sequences of the bacterium were found in nine animals, namely *Bandicota savilei*, *Berylmys bowersi*, *Leopoldamys edwardsi*, *Rattus exulans*, *R. tanezumi*, and *Rattus* sp. phylogenetic clade 3, yielding 2.3% infection rate, with two new rodent species found infected by the bacterium in Thailand: *B. bowersi* and *L. edwardsi*. Using a generalized linear mixed model (GLMM) and Random Forest analyses for investigating the association between human-land use and occurrence of the bacterium, forest habitat appeared as a strong explicative variable of rodent infection, meaning that *O. tsutsugamushi*-infected animals were more likely found in forest-covered habitats. In terms of public health implementation, our results suggest that heterogenous forested areas including forest-converted agricultural land, reforestation areas, or fallows, are potential habitats for *O. tsutsugamushi* transmission. Further understanding of population dynamics of the vectors and their hosts in these habitats could be beneficial for the prevention of this neglected zoonotic disease.

## 1. Introduction

The obligate intracellular bacterium *Orientia tsutsugamushi* (Rickettsiales: Rickettsiaceae) is the causative agent of scrub typhus in humans, mainly reported in the Asia-Pacific region, and sporadically in some other regions of the world [1,2]. Approximately one million cases of scrub typhus occur annually in Asia, with a 7% to 10% fatality rate if the patients are not treated sufficiently and early in the course of illness [3,4]. Chiggers, the six-legged parasitic larval stage of mites belonging to the family Trombiculidae, are the disease vectors carrying and transmitting the bacterial agent through their bites. *O. tsutsugamushi* is strictly confined to this mite family [5], and potentially develops a symbiotic relationship with its mite host [6,7,8]. Chigger species from the genus *Leptotrombidium* are the main vectors of scrub typhus in Asia [9,10]. *O. tsutsugamushi* was also reported in other trombiculid species [11] but there is still a lack of supporting evidence to prove their vectorial role in disease transmission, or whether they attack humans. In terms of vertebrate hosts apart from humans, small terrestrial mammals such as rodents (rats, mice and ground squirrels), insectivores and tree-shrews have been reported as hosts of *Leptotrombidium* and are infected by the bacterium. They play important roles in the ecology of chigger mites and thus for the epidemiology of the disease [12,13]. In addition, some other vertebrates such as bats, birds or reptiles, can be also hosts for chigger mites [14,15]. It was hypothesized that their feeding activity on a wide range of hosts may explain the huge diversification of strains of *O. tsutsugamushi* [9,13].

A large diversity of chigger mites can infect Asian small mammals, particularly rodents [16,17] and similarly, a large number of rodent and other small mammal species have been found to be infected by *O. tsutsugamushi* [18]. In Thailand, more than 10 species of rodents (*Bandicota indica, B. savilei, Berylmys berdmorei, Menestes berdmorei, Mus caroli, Niviventer fulvescens, Rattus andamanensis, R. argentiventer, R. exulans, R. losea, R. norvegicus* and *R. tanezumi*) and one species of tree-shrew (*Tupaia glis*) have been reported to be infected by the bacterium [12,19,20,21,22,23,24,25]. According to these studies, *O. tsutsugamushi* was detected using serological methods (i.e., fluorescence antibody assay), or by detecting the genetic material of the bacterium via specific gene target polymerase chain reaction (PCR) assays (i.e., the 56-kD type-specific antigen gene).

With the advent of modern molecular tools in bacteriology, high throughput sequencing provides affordable costs, effective detection, and rapid generation of microbial profiling using two main approaches: 16S rDNA amplicon sequencing (sequencing the genome of particular gene targets) and microbiome metagenomics (sequencing the whole particular genome in a sample) [26]. Here in the present study, 16S rDNA amplicon sequencing was conducted to screen the 16S rDNA gene of *O. tsutsugamushi* alongside other bacterial profiles in wild rodents from Thailand.

Environmental factors, including land cover and land use, are known to influence the reproduction and survival of both trombiculid mites [27,28] and rodents [29], and ultimately the spatial heterogeneity of infection risk. The increasing incidence of scrub typhus in South China was associated with habitats characterized by forested areas and climate factors such as relative humidity [30]. In Taiwan, a significant correlation was observed between scrub typhus incidence and habitats characterized by a mosaic of cropland and vegetation [28], which represent transitional land cover use. Scrub typhus occurs in transitional habitats such as forest edges, fallows along streams or abandoned agricultural lands [9]. These habitats favor both chigger mites, because of soil moisture, and their rodent hosts, by providing food and shelters [31].

The present study proposes to investigate the relationships between the structure of landscape and *O. tsutsugamushi* incidence in rodents captured from human-dominated habitats in Thailand. The structure of landscapes may promote *O. tsutsugamushi* transmission in rodents, and consequently the risk of transmission to humans [32,33]. Heterogenic landscape affects the distribution and abundance of rodent species regarding their specialization to habitat [29]. We explored the relationship between the structure of the landscape at a fine environmental scale and individual rodents investigated for infection by *O. tsutsugamushi*. For this, we used the land covers developed for investigating other rodent-borne diseases for each locality investigated in this study [29]. Specifically, we hypothesized that heterogeneous habitats with high forest cover may favor the transmission ecology of *O. tsutsugamushi* among rodents and mites, and its potential transmission to humans.

## 2. Materials and Methods

### 2.1. Ethical Statement

Rodent species included in the study are neither on the Convention on International Trade in Endangered Species of Wild Fauna and Flora (CITES) list, nor the Red List (IUCN). Animals were treated in accordance with the guidelines of the American Society of Mammologists, and within the European Union legislation guidelines (Directive 86/609/EEC). Each trapping campaign was validated by the national and local health authorities. Approval notices for trapping and investigation of rodents were provided by the Ethical Committee of Mahidol University, Bangkok, Thailand, number 0517.1116/661 (see [29] for more details), based on the CERoPath protocols for field and laboratory rodent studies [34].

### 2.2. Study Sites and Rodent Trappings

**Figure 1:**
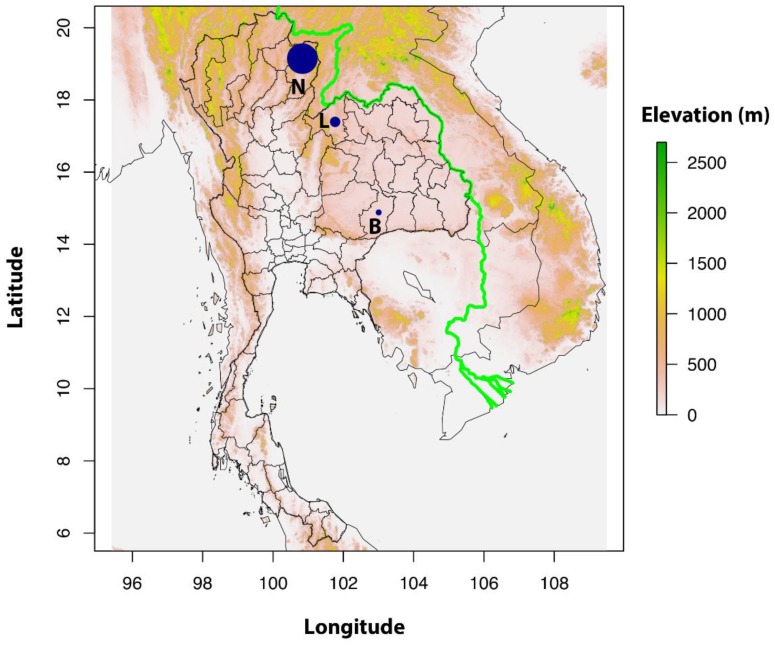
Map of Thailand with locations of the three sample sites in the provinces of Buriram (B), Loei (L) and Nan (N), with reported cases of scrub typhus per 100,000 from 2003–2007: 72.5 in Nan, 23.3 in Loei and <12.2 in Buriram. The relative size of the blue circles indicates average scrub typhus case numbers at each site. Nan and Loei belonged to the 10 leading provinces in the incidence rate of scrub typhus [35].

Three different sites in Thailand were investigated for the presence of *O. tsutsugamushi*. Rodents were trapped in the provinces of Buriram (14.89 N, 103.01 E), Loei (17.39 N, 101.77 E) and Nan (19.15 N, 100.83 E) in 2008 and 2009 (Figure 1). These sampling sites were part of the CERoPath project (www.ceropath.org). Reported human cases of scrub typhus were obtained from national statistics [35]. Reported cases of scrub typhus per 100,000 from 2003–2007 were 72.5 in Nan, 23.3 in Loei and less than 12.2 in Buriram. Nan and Loei belonged to the 10 leading provinces in the incidence rate of scrub typhus [35] (Bureau of Epidemiology, 2007).

The methodology was described in Morand et al. [29]. Trapping sessions were conducted in each locality in both wet and dry seasons during 2008–2009. At each locality, the same trap-lines were trapped over a four-night period during each trapping session, with 30 lines of 10 traps (10 m between each trap) placed in three different habitats, namely: (1) forests and mature plantations (rubber trees, teak trees, palm trees, coffee trees), (2) non-flooded lands or fields (shrubby waste land, fallow, young plantations, orchards), (3) rain-fed or irrigated lowland paddy rice fields corresponding to a cultivated floodplain (irrigation allows several crop harvests per year). This corresponded to a total of 1200 night-traps per trapping session. Locally-made live cage-traps were used, and the lines were placed in the same positions during each trapping session using GPS coordinates. Villages and isolated houses, which corresponded to a fourth habitat category, human settlement, were also sampled opportunistically using cage-traps distributed to residents.

Rodent and shrew species were identified in the field using morphological criteria, but were confirmed using molecular methods if needed using a mitochondrial gene for barcoding of some rodent species [36]. Complete data on the animals used as reference specimens for the barcoding assignment are available on the ‘Barcoding Tool/RodentSEA’ section of the CERoPath project web site (www.ceropath.org).

### 2.3. Environmental Indices and Land Use

The methodology was described in Morand et al. [30] and Bordes et al. [33]. For each locality, recent (years 2007–2008) high spatial resolution (2.5 m in panchromatic mode and 10 m in multispectral mode) SPOT 5 satellite images were acquired (CNES 2009©, distributed by Astrium Services/Spot Image S.A., Toulouse, France). SPOT-digital elevation model (DEM) with a spatial resolution of 20 m together with SRTM (shuttle radar topography mission) was also acquired. For each locality, the SPOT scene was classified into different land-cover types using an object-based approach. The land-cover maps and the DEM were integrated into a geographic information system (GIS) in order to compute landscape metrics for each trapping site. In order to describe the landscape surrounding the trapping location of each individual rodent, landscape metrics were calculated within a 100 m radius. These metrics included: cover of agriculture on steep land, cover of agriculture on flat land, cover of irrigated agricultural land, cover of forest, cover of human settlement, cover of irrigated land, a proxy of habitat diversity (patch density), a proxy of habitat fragmentation (edge density), and all distances between each rodent trapped and each land-cover types.

### 2.4. Rodent Screening for O. tsutsugamushi

Rodent spleens were placed in RNAlater^®^ storage solution (Sigma-Aldrich, Saint Louis, MO, USA) then stored at −20 °C until further analysis. Genomic DNA was then extracted from the spleen using the DNeasy® 96 Tissue Kit (Qiagen, Germany). Spleen DNA samples were screened for the presence of bacteria using universal primers (16S-V4F [GTGCCAGCMGCCGCGGTAA] and 16S-V4R [GGACTACHVGGGTWTCTAATCC]) targeting the hypervariable region V4 of the 16S rRNA gene (251 bp) via Illumina MiSeq (Illumina) sequencing. The V4 region has been proven to have excellent taxonomic resolution at the genus level [37]. A multiplexing strategy enabled the identification of bacterial genera in each individual sample. We followed the method described in Kozich et al. [38] to perform PCR amplification, indexing, pooling, de-multiplexing and finally taxonomic identification using the SILVA SSU Ref NR 119 database as a reference [39] (http://www.arb-silva.de/projects/ssu-ref-nr/). We then used the trimming strategy of Galan et al. [39,40] in order to clean the raw data set and to estimate reliable rodent positivity for bacteria.

### 2.5. Statistical Analyses

To analyze the infection of rodents, we first modeled the probability of presence/absence of *O. tsutsugamushi* as a function of several environmental indices with logistic regression, GLMM with logit function and random effects (locality), using package ‘lme4’ [40,41] implemented in R freeware [41,42]. The binomial variable, infected or non-infected individual rodent, was used in the logistic regression. The initial model included the environmental indices related to the habitat structure, and calculated for a buffer of 100 m for each individual rodent trapped: cover of agriculture on steep land, cover of agriculture on flat land, cover of irrigated agricultural land, cover of forest, cover of human settlement, cover of irrigated land, a proxy of habitat diversity (patch density), and a proxy of habitat fragmentation (edge density). No interactions were added among the independent variables. We evaluated support for competing models, investigating the relationship between the prevalence of *O. tsutsugamushi* and all explanatory variables of interest. We used AIC adjusted for sample size (AICc) to assess the relative information content of the models. We quantified the uncertainty that the ‘best’ model would emerge as superior if different data were used with Akaike weights (***w_r_***), the probability that a particular model is the best one from the available data. Selection of the best competing models was made using the package ‘glmulti’ (version 1.0.7 2) [42,43] implemented in R, which allows the exploration of all models using automated model selection and model-averaging procedure using a genetic algorithm. 

We also modeled the probability of presence/absence of *O. tsutsugamushi* as a function of several environmental indices with statistical learning Random Forest [43,44] using the ‘randomForest’ package [44,45]. The measures of importance of each variable are given by values of mean decrease accuracy, i.e., the decrease of model accuracy when the variable is dropped and by values of mean decrease Gini impurity, an index measuring the importance of each variable. All statistical analyses were performed in R freeware [40,41].

## 3. Results

### 3.1. Micro-Mammal Identification and O. tsutsugamushi Screening

In total, 387 rodents and shrews were captured and identified at species level (Table 1). The 16S rDNA sequences of *O. tsutsugamushi* were found in only nine rodent individuals (representing 2.3% rate of infection of all the micro-mammals investigated), i.e., *B. savilei* (2), *B. bowersi* (1), *L. edwardsi* (1), *R. exulans* (1), *R. tanezumi* (2), and *Rattus* sp. clade 3 (2) (Table 1). Two rodent species were newly recorded in Thailand for infection by the bacterium: *B. bowersi* and *L. edwardsi*. *O. tsutsugamushi* was detected in all the three localities: Buriram (5), Loei (3), and Nan (1) (Table 1).

### 3.2. Individual Surrounding Habitat Characteristics and O. tsutsugamushi Infection

Infected rodents were trapped in forested and reforestation areas, fallows, cassava plantations, and rice fields.

**Figure 2:**
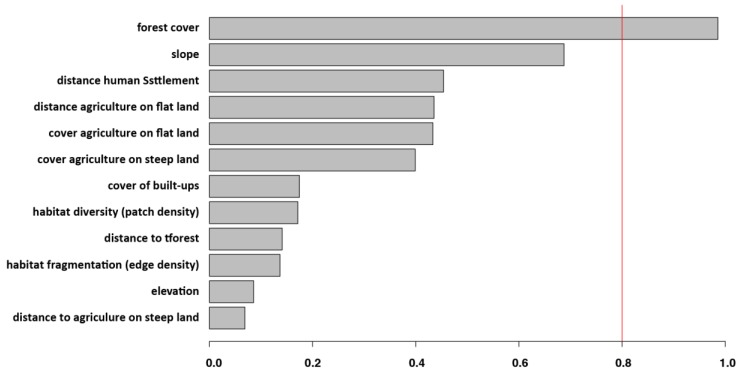
Results of GLMM model-averaged importance of surrounding habitat characteristics explaining the infection of rodents by *O. tsutsugamushi.* Note that only the explanatory variable forest cover is found in 100 per cent of the top best models.

Comparisons of models testing the effect of several surrounding habitat characteristics on individual rodent infection (GLMM with logit function) are given in Table 2 (best top 3 models) for the three sites in Thailand. Results of GLMM model-averaged importance of surrounding habitat characteristics explaining the infection of rodents by *O. tsutsugamushi* showed that only the explanatory variable forest cover is found in 100 per cent of the top best models (Figure 2). 

Hence, the best top model selected using AICc values demonstrated that surrounding habitat characterized by large forest cover on slow slope, distant from agriculture on flat land, explained the occurrence of the bacterium in rodents (Table 2). However, forest cover was the only significant variable (*p* < 0.05, Table 3).

**Figure 3:**
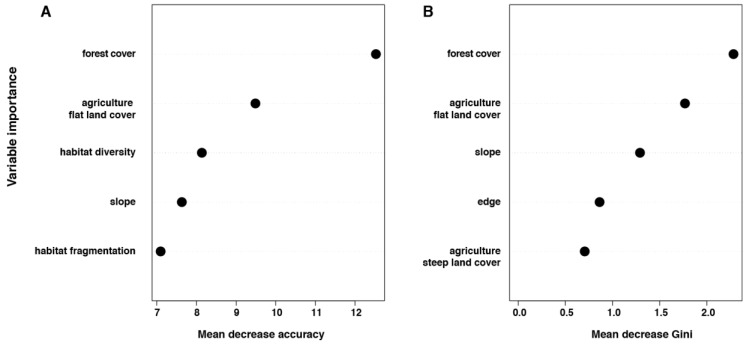
Results of Random Forest analysis with: (**a**) values of mean decrease accuracy; measuring the decrease of model accuracy when variables are dropped; (**b**) values of mean decrease Gini impurity index measuring the importance of each variable.

Using Random Forest analysis, again forest cover was selected as the best explanatory variable, followed by cover of agriculture on flat land (Figure 3). However, the values of mean decrease accuracy and mean decrease Gini dropped from cover of agriculture on flat land (Figure 2). Although the accuracy of the Random Forest was high with a value of 0.991 (with 95% CI: 0.97–0.999), the *p* value was not significant (*p* = 0.059), due to the low number of infected rodents (9) compared to the negative ones (378). 

## 4. Discussion

The goal of this study was to investigate the relationship between the landscape structure of the three localities and the occurrence of the bacterium *O. tsutsugamushi* in wild rodents detected using the 16S rDNA amplicon sequencing approach. Our findings indicate that the prevalence of the bacterium is very low, with only nine out of the 387 micro-mammals investigated, positive (2.3%). The prevalence of bacterium in animals was higher on the site of Buriram than the sites of Nan and Loei, in contrast to the human scrub typhus incidence report, which gave higher human incidence in Nan and Loei than in Buriram [35]. However, the data were not obtained at the same scale; the whole province is represented in health surveillance, versus only a few square kilometers for the rodent survey.

We confirmed the presence of the bacterium in several known rodent host species, such as *B. savilei, R. exulans* and the two species of the *R. tanezumi* complex [24]. We confirmed that the *Mus* species did not appear as infected by *O. tsutsugamushi* [21]. We recorded two new infected rodent species in Thailand: *B. bowersi* and *L. edwardsi*. The positive rodents are either habitat specialists, such as the forest-dwelling *L. edwardsi*, or habitat generalists, such as *R. tanezumi* [29]. Moreover, our results showed the epidemiological importance of the two synanthropic species, *R. exulans* and *R. tanezumi*, which live in close association with humans [29,46,47].

Our results seem to support our hypotheses that rodents infected by *O. tsutsugamushi* were more likely to be found in environments with large forest cover using either GLMM or Random Forest analysis. Our results suggest that a rodent was likely infected in a habitat such as a house, a fallow, or a rice field, if these habitats were in the vicinity of a forested area. The low support values (***w_r_***) for the overall models could be explained by the fact that the prevalence of the bacterium is very low with a low number of positive individuals for each land use land cover of the three localities. This low prevalence also explains why the accuracy of the Random Forest analysis was not significant (although close to significance).

The significance of forest cover for scrub typhus seropositivity was expected, as several studies conducted in Taiwan or in China showed that the incidence of scrub typhus is related to land use characterized by forested areas with abandonment of agriculture land and return to fallows and forests [28,30,31]. Southeast Asia is characterized by increased fragmentation and conversion of forest to agricultural land [48,49]). This new frontier of forest conversion is potentially risky habitat for scrub typhus infection.

### 4.1. Limitations of Our Study

There are some limitations of our study. First, we are just starting to obtain an evaluation on the sensitivity of 16S amplicon sequencing in the frame of epidemiological studies. Razzauti et al. [50] recently showed that the sensitivity of 16S rRNA amplicon sequencing on the MiSeq platform was equivalent to that of whole-RNA sequencing (RNA-seq) on the HiSeq platform for detecting bacteria in rodent samples. Galan et al. [36] revealed an excellent repeatability of bacterial detection (93%) using systematic replicates in 711 rodents. Finally, work in progress at our laboratory (unpublished result) shows that the sensitivity of the 16S rRNA amplicon sequencing approach used here is as good as the sensitivity of quantitative PCR (qPCR) performed with specific primers, the current gold standard for bacterial detection in biological samples. Thus, we are confident in our *Orientia* screening.

Second, more detailed information on human cases at high resolution, i.e., at the level of sub-district or villages, will help to accurately investigate accurately the relationship between human cases and rodent infection by *O. tsutsugamushi*.

Third, our study could not assess the impact of seasonality on the epidemiology of *O. tsutsugamushi*. Another limitation concerns the diversity and infection of chigger mites. All chigger mites were collected and preserved. Future studies are planned to explore this point.

### 4.2. Implications for Public Health

The incidence of scrub typhus is increasing in several countries of East Asia. Previous studies [30] showed that habitat fragmentation and extensive agriculture disrupt rodent habitats favoring generalist and synanthropic rodent species, which ultimately enhance rodent-borne transmission. The results of our study suggest that risky habitats for *O. tsutsugamushi* transmission are heterogenous forested areas, comprising conversed agriculture or reforested areas, fallow or abandoned agriculture. Surveillance of rodents’ population dynamics in these habitats may help to prevent zoonotic transmission.

## Figures and Tables

**Table 1:** List and number of host species infected by *O. tsutsugamushi* in three localities of Thailand.

Locality	Micro-Mammal Species	Number Tested	*O. tsutsugamushi* Positive
Buriram	*Bandicota indica*	2	0
	*Bandicota savilei*	10	2
	*Menetes berdmorei*	1	0
	*Mus caroli*	8	0
	*Mus cervicolor*	11	0
	*Mus cookii*	1	0
	*Rattus argentiventer*	1	0
	*Rattus exulans*	32	0
	*Rattus sakaretensis*	2	0
	*Rattus tanezumi*	3	1
	*Rattus sp. clade 3*	26	2
Loei	*Bandicota indica*	15	0
	*Bandicota savilei*	12	0
	*Berylmys berdmorei*	9	0
	*Berylmys bowersi*	14	1
	*Cannomys badius*	1	0
	*Chiropodomys gliroides*	2	0
	*Crocidura attenuate*	1	0
	*Hapalomys delacouri*	1	0
	*Leopoldamys edwardsi*	5	1
	*Leopoldamys sabanus*	5	0
	*Maxomys surifer*	15	0
	*Mus caroli*	5	0
	*Mus cervicolor*	5	0
	*Mus cookii*	6	0
	*Mus fragilicauda*	1	0
	*Niviventer fulvescens*	16	0
	*Rattus exulans*	26	1
	*Rattus sakaretensis*	30	0
	*Rattus nitidus*	1	0
	*Rattus tanezumi*	16	0
	*R. tanezumi clade 3*	2	0
Nan	*Bandicota indica*	30	0
	*Berylmys berdmorei*	8	0
	*Berylmys bowersi*	1	0
	*Mus caroli*	1	0
	*Mus cookiie*	4	0
	*Rattus exulans*	24	0
	*Rattus tanezumi*	25	1

**Table 2:** Comparison of models testing the effect of several surrounding habitat characteristics on individual rodent infection by *O. tsutsugamushi* [generalized linear mixed model (GLMM) with logit function] for the three localities (locality as random factor). Models are ranked from lowest to highest supported, according to corrected Akaike information criteria (AIC). Only localities with at least one individual infected rodent were kept for each analyzed dataset. The initial model included the following explanatory variables: cover of agriculture on steep land, cover of agriculture on flat land, cover of agriculture on irrigated land, cover of forest, cover of human settlement, habitat diversity (patch density), habitat fragmentation (edge density), slope, distance to agriculture on flat land, distance to agriculture on steep land, distance to forest, distance to human settlement (K is the number of estimated parameters, AICc is the selection criterion, and ***w_r_*** are the Akaike weights)

Models (Best Top Three)	K	AICc	*w_r_*
slope + cover of forest + distance to agriculture on flat land	4	84.70	0.033
slope + cover of forest	3	85.02	0.028
Slope+ cover of agriculture on steep land + cover of agriculture on flat land + cover of forest + distance to agriculture on flat land + distance to human settlement	6	85.22	0.025

**Table 3:** Results of the best GLMM (with logit link function, and locality as random factor) explaining the occurrence of *O. tsutsugamushi* in rodents, as a function of surrounding habitat characteristics (see Table 2 for the initial models and best top 3 selected models) (estimate of the logit function with SD = standard deviation, residual deviance with DF = degree of freedom).

Explanatory Surrounding Habitat Characteristics	Estimate (SD), *p*	Log Likelihood, Deviance (DF)
Forest cover	3.87 (1.25), 0.002	
Distance to agriculture on flat land	0.003 (0.001), 0.096	
Slope	−0.22 (0.14), 0.10	−160.6, 321.1 (1360)
